# Feasibility and acceptability of peer‐delivered HIV and syphilis self‐test kits and assisted partner notification services for gay, bisexual and other men who have sex with men: a qualitative study in Uganda

**DOI:** 10.1002/jia2.26456

**Published:** 2025-05-19

**Authors:** Stephen Okoboi, Andrew Mujugira, Deborah Ekusai‐Sebatta, Adeline Twimukye, Peninah Tumuhimbise, Brian Aliganyira, Barbara Castelnuovo, Rachel King

**Affiliations:** ^1^ Infectious Diseases Institute Makerere University Kampala Uganda; ^2^ Department of Global Health University of Washington Seattle Washington USA; ^3^ Infectious Diseases Research Collaboration Makerere University Kampala Uganda; ^4^ Ark Wellness Hub Kampala Uganda; ^5^ Department of Epidemiology and Biostatistics Institute for Global Health Sciences University of California San Francisco San Francisco California USA

**Keywords:** HIV self‐testing, syphilis self‐testing, assisted partner notification, GBMSM, healthcare workers, Uganda

## Abstract

**Introduction:**

There is a need to combine different approaches to tackle the HIV epidemic, particularly in high‐incidence populations. We explored the feasibility and acceptability of using peer‐delivered HIV self‐testing (HIVST), syphilis self‐testing (SST) and assisted partner notification (APN) services among gay, bisexual and other men who have sex with men (GBMSM) in Uganda.

**Methods:**

From November 2023 to March 2024, we conducted in‐depth interviews with 20 purposively selected GBMSM peers and 10 healthcare workers (HCWs). The GBMSM and HCWs interviews explored their perspectives on (1) the feasibility, acceptability and preferences for peer‐delivered interventions (HIVST, SST and APN) and (2) strategies and methods of reaching individuals who had not been tested or tested more than 6 months before the interview. We used a content analysis approach to define and organize codes deductively and inductively to identify themes.

**Results:**

The median age of the 20 GBMSM peers was 27 years (interquartile range [IQR], 22–35 years), and 37 years (IQR, 25–52) for the 10 HCWs, of whom seven were female. We identified four emerging categories: (1) *Trust*: GBMSM peers and HCWs expressed trust in the peer delivery of self‐test kits (HIVST and SST) to obtain same‐day results effectively. HCWs were preferred over peers for APN services in reaching sexual contacts of index clients for testing; (2) *Intimate partner violence (IPV)*: Although initial concerns about IPV were raised concerning both HIVST programmes and peer APN strategies, such incidents were rarely reported in practice; (3) *Entry point*: Similar to HIVST, SST was a self‐administered activity that served as an entry point for HIV testing discussions among GBMSM who had either never undergone or had postponed testing. Self‐test kits could also facilitate pre‐sexual testing among GBMSM; (4) *Social media*: Campaigns on social media dedicated to promoting self‐testing could expand testing coverage services to GBMSM vulnerable to HIV and syphilis acquisition.

**Conclusions:**

HCWs and GBMSM peers preferred delivery of self‐test kits through peers over facility‐based approaches; however, they favoured HCWs for providing APN services. Integrating peer‐delivered self‐testing programmes into differentiated testing models and leveraging social media influencers could expand testing coverage among GBMSM.

## 1 INTRODUCTION

Globally, gay, bisexual and other men who have sex with men (GBMSM) have 28 times greater probability of exposure to human immunodeficiency virus (HIV) acquisition than adult heterosexual men (15−49 years) in the general population [1, 2]. Syphilis and HIV disproportionately affect GBMSM because of sexual network dynamics that include the probability of exposure to concurrent sexual partnerships [3]. HIV and syphilis co‐infection is common because syphilis and HIV have similar modes of transmission [3, 4].

In sub‐Saharan Africa, 6% of new HIV acquisitions occurred among GBMSM in 2021 [1]. In Uganda, HIV prevalence among GBMSM is estimated at 13.2% compared to 4.7% in similarly aged males [5, 6, 7]. Syphilis prevalence among GBMSM in Uganda is estimated at 9.0% compared with 3.6% in males aged 15–64 years [5, 8]. Scaling up HIV/syphilis testing is critical to reaching undiagnosed GBMSM living with HIV [9, 10] achieving the World Health Organisation (WHO) Global Health Sector Strategy goal of reducing syphilis incidence by 90% by 2030 [3] and the first United Nations Programme on HIV/AIDS (UNAIDS) 95‐95‐95 goal of 95% of persons with HIV knowing their status by 2025 [8]. However, current testing strategies in sub‐Saharan Africa only reach men with a lower likelihood of HIV acquisitions and are already tested for HIV and are thus low‐yield [6]. Therefore, identifying high‐yield strategies for dual HIV/syphilis testing that effectively reach GBMSM in high‐incidence sexual networks is critical [7]. Decentralized self‐administered HIV self‐testing (HIVST) and syphilis self‐tests (SSTs) approved by WHO permit the user to interpret test results by themselves in private. They could improve dual HIV/syphilis test uptake in this population. The WHO recommends peer approaches to HIV testing [11, 12], which can be leveraged to increase coverage of dual HIV/syphilis. Combining HIVST and SST with peer delivery offers a unique opportunity to reach GBMSM where they are with user‐friendly technology. Peer delivery is a person‐centred approach [13] that could maximize the coverage, effectiveness, efficiency and impact of HIV/syphilis services in Uganda.

Our prior work in Uganda found that peer‐delivery of HIV self‐tests was acceptable to 90% of GBMSM in our study [14, 15]. The WHO recommends SST as an additional approach to syphilis testing services [12]. Co‐packaging HIVST with SST and ensuring linkage to confirmatory testing and immediate treatment initiation reflects global guidance aimed at providing integrated care [11, 12]. This includes partner notification, comprehensive HIV/sexually transmitted infections (STI) testing and treatment, expedited partner therapy for STI management, HIV pre‐exposure prophylaxis (PrEP) and doxycycline post‐exposure prophylaxis for STI prevention [12]. Thus, HIVST and SST linked with partner services may empower and expand testing uptake and coverage [16].

Assisted partner notification (APN) services recommended by the WHO are the notification of sexual partners of those with a probable exposure to HIV acquisition [3]. APN is acceptable and feasible [17, 18, 19, 20], increases testing uptake among sexual partners [17, 20, 21], enables early diagnosis and linkage to care and other prevention services, motivates behaviour change in index participants (i.e. people living with HIV) and their partners, and may reduce STI and HIV burden [17, 18, 19, 20]. Thus, APN could efficiently identify GBMSM with undiagnosed HIV and syphilis infection. A pilot APN programme in rural Uganda that enrolled 464 people with HIV and tested their recent sexual partners found that 32% (61/193) of sexual partners who were traced and tested were diagnosed with HIV [21]. A combination of APN, HIVST and community worker outreach was acceptable and feasible in Kenya [22] and increased uptake of HIV testing and linkage to care and PrEP among GBMSM and their sexual partners [22, 23]. A systematic review of 26 trials conducted globally found that APN identified more undiagnosed syphilis cases than passive notification [24]. The WHO also recommends peer‐led demand‐creation interventions to increase HIV/syphilis test uptake and to engage those in greatest need of services [12]. Integrating peer‐delivered HIV/syphilis testing with APN could help expand access to testing for HIV and syphilis testing; however, this strategy has not been evaluated among GBMSM in Uganda. Our study aimed to evaluate the feasibility and acceptability of providing combined peer‐delivered HIV and SST kit distribution alongside APN services for Ugandan GBMSM.

## 2 METHODS

### 2.1 Population and procedures

We conducted formative research from November 2023 to March 2024 in preparation for a cluster‐randomized trial to pilot‐test the preliminary effectiveness of peer‐delivered HIV/SST and APN services versus facility‐based testing. The intervention comprises peers (1) delivering HIVST/SST kits to MSM within their networks using social networks and targeting never or infrequent testers; (2) actively linking GBMSM who test positive to confirmatory testing and care; (3) offering sexual partners voluntary APN and HIV/syphilis testing. This study was conducted with GBMSM peers (aged 18 and older) and healthcare workers (HCWs) who provide care to members of key populations (KPs) in Kampala and Wakiso districts, located in central Uganda. These districts are part of a larger network of 200 KP peers nationwide supported by the President's Emergency Plan for AIDS Relief, a funded programme administered by Makerere University Infectious Diseases Institute (IDI). These peers are assigned to high‐volume KP facilities and drop‐in centres, also known as “safe spaces” or social environments, which facilitate meaningful peer involvement, offer health‐related services, and primarily focus on creating demand for and distributing HIVST kits within their social networks.

The Ark Wellness Hub (an MSM‐led organization), IDI KP healthcare Programme Officers and Principle Investigator (PI) mobilized the peers. They purposively identified their peers in Kampala and Wakiso districts who support KP programming. The project PI purposively identified HCWs in liaison with IDI's KP programme advisor and Ark Wellness focal person. Peer inclusion criteria were: (1) having >6 months of self‐test experience and kits distribution; (2) aged >18 years; (3) residing in the study catchment area (Kampala and Wakiso); (4) having experience with demand creation for testing services; and (5) assigned to a drop‐in centre, a health facility or a community venue managed by the IDI. The author SO purposefully included HCWs who had provided KP programme activities in Kampala and Wakiso districts for at least 3 years; this was deemed sufficient experience in KP programming and service delivery. The KP Programme Officer and Ark Wellness focal person introduced the study team to the peers, while the KP Programme Adviser introduced the study team to the HCWs. The author SO contacted HCWs by phone to explain the study. Twenty in‐depth interviews were conducted with GBMSM peers and 10 with HCWs to gather their perspectives on (1) the feasibility and acceptability of peer‐delivered intervention that include HIVST, SST and APN; (2) preferences for HIV/syphilis testing and APN approaches; and (3) effective strategies for reaching individuals who had not been recently tested or tested more than 6 months before the interview.

### 2.2 Data collection

We conducted a pilot test of the GBMSM peer and HCW interview guides with two peers and two HCWs, whom we excluded from the final sample, at ART Wellness Hub, a drop‐in centre under the supervision of author RK. A key revision recommended after pilot testing the tools were: (1) eliminating the word “recruit” from questions like “What approaches do you recommend for recruiting fellow GBSMSM who have not tested before?” We revised it to “What approach do you recommend for reaching those who have not tested before for HIV and syphilis?” The word “recruit” is punishable by law as it is interpreted to mean recruiting people to become GBMSM according to the Uganda Anti‐Homosexuality Act 2023; and (2) incorporating person‐centred words that convey stigma‐free expressions to the interview guides. The domains (preference, feasibility and acceptability) were similar for HCWs and peer interview guides. Interviews were conducted in a private room at the IDI Mulago campus and Ark Wellness Hub, where conversations could not be overheard for GBMSM and virtually via Zoom or in person for HCWs. Participants were given the option to decline participation in the study. The number of participants interviewed provided sufficient information to reach saturation of themes. The interviews were conducted by an experienced cisgender social scientist (authors DE‐S and PT) and a cisgender male epidemiologist (author SO), who is experienced in qualitative data collection. Each interview lasted 48–65 minutes and was audio‐recorded in English or Luganda, the local language in Central Uganda. Luganda interviews were translated and transcribed into English by an independent person experienced in transcription. Acceptability was defined as stakeholders’ perceptions that peer‐delivered HIV and SST and the APN approach are agreeable, palatable or satisfactory. Feasibility was the appropriateness of the peer‐delivered HIV and SST and APN intervention for further evaluation. Preference was defined as participants’ interest in either peer‐delivered HIV/SST or a facility‐based HIV and SST approach.

### 2.3 Data analysis

We used a deductive and inductive content analytic approach to define and organize codes and identify themes [25]. We used NVivo 11 software (QSR International, Melbourne, Australia) for systematic data management [25]. The data coding proceeded as follows: (1) authors SO and AT independently read all interview transcripts and field notes, and identified major codes. The independent analysis helped to ensure that those involved did not influence each other during the interpretation of the data; (2) a selection of transcripts was double‐coded and subsequently compared. Any discrepancies were resolved in iterative discussions, resulting in a codebook that included descriptive labels for each coded category, (3) we developed codes to capture categories that emerged in the data using the constant comparison method. The analysis continued iteratively, refining the major categories and codes via discussion between SO and AT. Quotes that illustrated each emerging theme were chosen to present the results. We used the Consolidated Criteria for Reporting Qualitative Research Findings (COREQ) to report study findings [26].

### 2.4 Ethics approval

This study was approved by the Infectious Diseases Institute Research Ethics Committee (IDI‐REC‐2023‐51) and the Uganda National Council for Science and Technology (HS3021ES). Participants provided written informed consent in English and Luganda.

## 3 RESULTS

### 3.1 Participants characteristics

The median age of the 20 GBMSM peers interviewed was 27 years (interquartile range [IQR] 22–35) and 37 years (IQR 25–52) for the 10 HCWs, with seven of them being cisgender female. Most peers (70%) were working as peers at drop‐in centres and community venues, and less than half (45%) had attained tertiary education. Half of the HCWs interviewed (50%) had a Master's degree, the highest education level attained, and half worked as KP Focal Persons (Table 1). Two peers declined to participate because of safety concerns following the enactment of the 2023 Anti‐Homosexuality Act in Uganda [29] and were subsequently replaced.

**Table 1:** Demographic characteristics of participants enrolled in the study in Uganda

Variable	*N* (%)
**Peers *N* = 20**	
**Age**	
20−29	14 (70)
30−39	6 (30)
**Age, years** (median, IQR)	26.7 (IQR = 22−35)
**Educational level**	
Degree level	6 (30)
Tertiary level	9 (45)
Secondary level (14 years of education)	5 (25)
**Occupation**	
Peers at the government health facility	3 (15)
Peers at drop‐in centre/or community venues, club	14 (70)
Peers at private civil society HIV clinic	3 (15)
**Healthcare workers *N* = 10**	
**Age**	
20−29	3 (30)
30−39	4 (40)
>40	3 (30)
**Age, years** (median, IQR)	37.3 (IQR = 25−52)
**Gender**	
Female	7 (70)
Male	2 (20)
Transgender woman	1(10)
**Educational level**	
Masters	3 (30)
Bachelors	5 (50)
Tertiary level	2 (20)
**Occupation**	
Programme Coordinators/Officer	4 (40)
Key Population Focal Persons	5 (50)
Community Liaison Officer	1 (10)

### 3.2 Qualitative results

We present four categories to illustrate the feasibility and acceptability of implementing a peer‐delivered intervention for distributing HIV and syphilis self‐test kits and providing partner services to Ugandan GBMSM. First, GBMSM peers and HCWs expressed trust in peer delivery of self‐test kits (HIVST and SST) as an effective method for obtaining same‐day test results. HCWs were preferred over peers for APN services in reaching sexual contacts of index clients for testing. Second, initial concerns about intimate partner violence (IPV) were reported for both HIVST programmes and peer APN strategies, but were not observed in practice. Third, SST, like HIVST, was a self‐administered activity that served as an entry point for discussing HIV testing with GBMSM who had either never been tested or had delayed testing. Self‐test kits could also facilitate pre‐sexual testing among GBMSM. Finally, social media campaigns promoting self‐testing could expand the coverage of testing services to GBMSM with a higher likelihood of HIV and syphilis acquisition or transmission.

### 3.3 Category 1: GBMSM and HCWs trusted peer‐delivered self‐test kits to facilitate same‐day access to test results

GBMSM peers and HCWs in Central Uganda reported that GBMSM would have a high level of trust in the peer‐delivered HIVST and SST strategy. Peers expressed a willingness to distribute peer self‐testing kits and felt that this approach was feasible, acceptable and trustworthy because it allowed them to share personal experiences with one another. Additionally, the addition of the syphilis self‐test kit to the current package of HIV prevention commodities, including condoms and lubricants, was deemed to be easily achievable, as peers were already distributing them. This approach would facilitate access to syphilis and HIV testing, with immediate access to test results and rapid test interpretation facilitated by peers if so desired by the tester.

“Peer‐to‐peer, they will not be worried; they are on the same level. ‘This is my fellow; let us work together’. Most of the time, from what we have seen, they will even trust us to help them do the tests. So even with the results, the peer could get to know the results before they leave.” (HCW, age 39).

“It will be easy to use combined self‐test kits we [peers] have been distributing HIV self‐testing kits and other commodities; adding syphilis self‐test kit now will not be burdensome because many community members have syphilis [and] they don't know it.” (Peer, age 27).

### 3.4 Attitudes towards APN

GBMSM, who received a positive HIV test result, indicated a negative attitude towards peer APN and preferred the HCW APN approach over the peer APN approach due to confidentiality concerns expressed during peer HIV distribution. In instances where peers identified GBMSM who tested positive and their sexual partners, there was a probability of revealing the index person's HIV status to their sexual network members on GBMSM‐dedicated social media groups. Peers and HCWs said this information was personal, sensitive and intimate. Disclosing such information to members of one's sexual network could lead to emotional distress, including feelings of isolation and rejection from both network members and other GBMSM individuals.

“Yeah, GBMSM are not okay with assisted partner notification by peers because [they believe that] most peers share the information on their social media groups, and you will be isolated… the best way to me would be to go straight to the partner or health worker and invite the partner to a health facility. Health‐related issues are usually personal initiatives; you can't force someone to get what they need.” (Peer, age 25).

The HCWs APN approach was the preferred method for addressing these concerns, as providers were equipped with the necessary training and had established high trust in clients’ medical information. Confidentiality was also strictly upheld to prevent the identification of an index who had revealed their sexual contacts. Additionally, HCWs provided detailed instructions on how to conduct notification and provided clear explanations of the benefits of eliciting and testing sexual contacts.

“If I talk to a health worker and I pass on the partner's telephone number, my partner will not know that I'm the one who gave the number to the health worker, so it will be easier for me. When I give the health worker the number, they will call my partner and then encourage the partner to go for a test or contact them for a test. And there, if he tests positive, medication will be next so that it will be easier.” (Peer, age 25).

### 3.5 Category 2: Concerns about IPV were reported with HIVST and APN strategies

Peers and HCWs perceived the fear of IPV as a significant obstacle to implementing a GBMSM peer‐delivered HIV and SST self‐testing programme. There were concerns that GBMSM individuals would be pressured into receiving test kits against their free will, which would violate the principle of autonomy. However, HCWs and peers reported that IPV resulting from peer‐delivered self‐test kits was rarely reported in practice. The few cases reported were used as opportunities to improve communication when disclosing one's test results to an intimate partner by HCWs. In the experience of both groups of participants, partner notification was more effective in identifying individuals living with HIV compared to other testing approaches.

“We were worried about the intimate partner violence that was going to take place. We even started capturing that information, but we received few cases …. [The few cases help] you learn how to strategise and communicate. So, positivity rates are greater when we use APN.” (HCW 05, age 39).

“Domestic violence mostly comes with other issues, maybe drugs, some other cultural stuff; that's the main thing that causes violence, yeah, because some of us use a lot of drugs.” (Peer 16, age 24).

### 3.6 Category 3: HIV and SST was a self‐administered activity that facilitated pre‐sex testing

Peers highlighted that the integrated peer HIV and syphilis self‐test kits delivery would facilitate timely access and regular testing services among GBMSM vulnerable to HIV and syphilis acquisition when they tested and shared results before having sexual contact. HCWs explained that testing before sex enabled the uptake of appropriate prevention measures that limited the transmission to concurrent partners. It also addressed barriers to concurrent partner testing at health facilities since testing as partners at health facilities was a slow, lengthy process that did not facilitate timely, pre‐sex testing.

“Also, for people, especially GBMSM, ‘you know what, before we have sex, we first test. It's so easy before sex or before anything; it's easy for, like, ‘Okay, there is an [HIV self‐test] kit there. Let's just test before we go into anything. Going to a facility is a process, and by the time you go through that process, there is mistrust, disinterest.” (Peer, age 27).

HIV and SST were described as a self‐monitored activity where an individual decided to test themselves while carefully following the testing instructions and interpreting the results. Self‐testing facilitated self‐care and management, as the person could share the test results with peers.

“They [HIV and syphilis self‐test kits] are embraced mostly by GBMSM because you can use them wherever you are… everywhere they can be used, and they are self‐monitored activities. Someone sits somewhere alone, and they take it themselves. They test, and then they produce the results.” (HCW, aged 51).

“The purpose of the HIV and syphilis self‐kit is to test in private, in the comfort of your home, without the stigma attached to a government facility.…. So, I feel like the kit should be an opening into a person's life. So, you give them the kit and tell them, ‘Go home and test. You don't have to tell me the results, but let's keep talking.” (Peer, aged 27).

### 3.7 Category 4: Leveraging social media to encourage HIV and SST could increase prevention uptake

Leveraging social media to create demand and marketing HIV and SST programmes could increase the uptake of testing services. However, for participants who test positive for HIV and syphilis, distributing anonymous text messages was the preferred method for disclosing potential HIV or syphilis exposure to sexual network members of an index client. This approach ensured confidentiality and minimized fear within GBMSM communities. Additionally, the anonymity of the sender's details eliminated any suspicion among sexual network members, allowing them time to reflect on the message. By encouraging individuals to seek more information and potentially get tested, this method was perceived to promote open, non‐threatening communication, catalyse discussions about sexual behaviours, and encourage individuals to seek additional knowledge and consider getting tested.

“As compared to phone calls, text messages through applications have proved to be more effective because they are less frightening to the community: ‘Hi X, one of your sexual partners has tested positive for HIV. You could consider passing by this facility to get tested. Alternatively, if you need more information, please call us at this number’. These messages are anonymous and do not show who sent them.” (Peer, aged 23).

Increasing targeted HIV and syphilis awareness campaigns through social media platforms by GBMSM social influencers could safely increase access to and supply testing services through online ordering and information sharing. This would enable their followers to expand the reach and usage of testing services, facilitating the uptake of testing services to those who had never tested or took a long time to test, as many GBMSM trusted their social media influencers.

“Identifying people in these communities who are influential in providing this information on social media platforms such as Twitter, WhatsApp, Facebook, and TikTok, they can use those platforms to reach that audience. These kits are available. They are free of charge; you can get them from here by sharing contact information on how to access them online.” (Peer, aged 23).

Using digital support to supplement in‐person services was crucial in expanding self‐testing capabilities in today's digital landscape. It had the potential to engage vulnerable populations and promote consistent HIV and SST services, fostering ongoing participation and proactive management of their health. Implementing a digital peer model could enable testing services to effectively engage individuals previously marginalized, particularly working‐class members, by overcoming constraints associated with traditional testing methods in an environment where belonging to a KP was criminalized.

“I think the whole peer model should be transformed for the digital age to provide access to more people having concurrent sexual partnerships. There is an opening to meet him in those spaces where he is with other queer people. So how do we provide services that are for ‘the working class’ of a typical gay, bisexual, and queer man? We have failed to reach them maybe because of the current laws, but also because people have not thought of those models.” (Peer, aged 27).

Leveraging M‐health to create demand and market the integrated peer‐delivered HIVST, SST and APN could expand testing uptake to people who are underserved and experiencing discrimination.

## 4 DISCUSSION

This study, conducted among GBMSM and HCWs, assessed the feasibility and acceptability of using peer‐delivered HIV and syphilis self‐test kits as well as APN services for addressing testing inequities among GBMSM with a greater likelihood of HIV and syphilis acquisition in Central Uganda. The findings indicated that combining peer delivery of these self‐test kits with APN services was feasible and acceptable for GBMSM in Uganda. GBMSM trusted peers and HCWs to deliver HIVST and SST, allowing for same‐day test results. While HCWs were preferred over peers for delivering APN services to sexual contacts of index clients, initial concerns regarding IPV following HIVST and APN were infrequently reported in practice. Similar to HIVST, SST was also a self‐administered activity that served as an entry point to care, enabling testing before engaging in sexual activity. GBMSM peers suggested using social media platforms to expand the coverage and reach of HIV and SST services within their community.

To our knowledge, this is the first qualitative evaluation of combined peer delivery of self‐test kits (SST and HIVST) and APN for African GBMSM. Our finding that peer‐delivered HIV and syphilis self‐test kits distribution and APN services for Ugandan GBMSM were feasible and trusted is comparable to previous studies done in Uganda [14], China [9] and Zimbabwe [27]. They found that peer distribution of HIVST and SST was feasible and effective in reaching social and sexual network members with testing services. Similarly, a qualitative study among GBMSM in Uganda, which interviewed 74 GBMSM, found that peer distribution of HIVST was feasible and acceptable [28]. Our finding that SST acted as an entry point to discussing HIV testing with GBMSM is similar to a Chinese study that found that SST was a key entry point for HIVST among GBMSM [9]. In this study, 92.5% of GBMSM social network members who received SST performed HIVST after receiving test kits and had access to same‐day test results [9]. These data support the scale‐up of dual HIV/SST for GBMSM.

Our findings suggest that implementing a digitalized peer delivery self‐testing model and targeted social media campaigns led by respected and influential members of the GBMSM community could expand testing services to this underserved group of men. This is supported by previous studies that have shown that distributing self‐testing and HIVST kits to Chinese GBMSM through social media effectively reached sub‐populations who may not have otherwise tested for HIV [29]. However, challenges still exist in verifying self‐test results [29]. In response, WHO recommended using digital support technology in 2019 as an innovative means to expand HIVST [30, 31]. Digital support encompasses a range of applications and web‐based messaging platforms designed to guide individuals through the testing process, aid in interpreting their results, and connect those who test positive with confirmatory testing and care options while linking those who test negative with HIV prevention services [29]. Globally, there is evidence that self‐tests are user‐friendly based on numerous studies [32, 33]. Future studies should evaluate digital marketing strategies on social media aimed at promoting HIV testing for underserved men.

GBMSM preferred that HCW implement partner notification services for the delivery of HIV and syphilis notifications. This approach ensures confidentiality for the index GBMSM, as there is a probability of their identity and HIV/syphilis status being shared if the peer is part of their sexual network. Furthermore, misunderstandings between the peer and index client could result in discussing sensitive information. This could lead to emotional distress for the index participant, including feelings of isolation and rejection from their network. HCWs have specialized training to protect and support the index participant through counselling and management of discussing HIV status to their sexual partners while also maintaining their anonymity.

Our study has several strengths, including being the first qualitative assessment of GBMSM peer‐delivered HIVST, SST and APN services in Uganda after the enactment of the Uganda Anti‐Homosexuality Act 2023 [34]. Using qualitative data allowed a comprehensive understanding of how best to implement integrated HIVST/SST delivery by peers. Additionally, two of our authors (BA and TP) are experienced with GBMSM populations and provided valuable insights into the project design, data collection tools design and interpretation of the data. A limitation of this study is that we only interviewed GBMSM older than 18 years, so our results may not be relevant to those under 18 years. Nevertheless, they can provide valuable insights for peer delivery of HIVST, SST and APN services to GBMSM in Uganda and elsewhere.

## 5 CONCLUSIONS

HCWs and GBMSM peers preferred delivery of self‐test kits through peers over facility‐based approaches; however, they favoured HCWs for providing APN services. Although IPV was initially considered a potential barrier to implementing peer‐delivered self‐testing initiatives, such incidents were infrequent in practice. Integrating peer‐delivered self‐testing programmes into differentiated testing frameworks and leveraging social media to promote HIV and syphilis self‐tests could expand prevention coverage among GBMSM populations.

## COMPETING INTERESTS

No conflicts of interest are to be declared.

## AUTHORS’ CONTRIBUTIONS

SO, AM and RK conceptualized the manuscript. SO, BA, PT and DE‐S conducted qualitative interviews under RK's supervision. SO and AT coded the data and then developed categories using an inductive approach. SO wrote the first draft with contributions from AM, RK and BC. AM, RK, DES, AT, PT, BA and BC reviewed drafts and made significant revisions. All authors read and approved the final version of the manuscript.

## FUNDING

This work was funded by a research grant from the National Institutes of Health, Fogarty International Center (K43TW012637, awarded to SO).

## DISCLAIMER

The opinions expressed in this paper are solely those of the authors and do not necessarily reflect the official views of the National Institutes of Health.

## Data Availability

Data and code book are available on request.
